# Deoxynivalenol & Deoxynivalenol-3-Glucoside Mitigation through Bakery Production Strategies: Effective Experimental Design within Industrial Rusk-Making Technology

**DOI:** 10.3390/toxins7082773

**Published:** 2015-07-24

**Authors:** Silvia Generotti, Martina Cirlini, Alexandra Malachova, Michael Sulyok, Franz Berthiller, Chiara Dall’Asta, Michele Suman

**Affiliations:** 1 Department of Food Science, University of Parma, Parco Area delle Scienze 95/A, Parma 43124, Italy; E-Mails: silvia.generotti@nemo.unipr.it (S.G.); martina.cirlini@unipr.it (M.C.); chiara.dallasta@unipr.it (C.D.); 2 Christian Doppler Laboratory for Mycotoxin Research and Center for Analytical Chemistry, Department IFA-Tulln, University of Natural Resources and Applied Life Sciences, Vienna, Konrad Lorenzstr. 20, 3430 Tulln, Austria; E-Mails: alexandra.malachova@boku.ac.at (A.M.); michael.sulyok@boku.ac.at (M.S.); franz.berthiller@boku.ac.at (F.B.); 3 Barilla G.R. F.lli SpA, Advanced Laboratory Research, via Mantova 166, 43122 Parma, Italy

**Keywords:** deoxynivalenol, deoxynivalenol-3-glucoside, design of experiment, food processing, baking, rusks

## Abstract

In the scientific field, there is a progressive awareness about the potential implications of food processing on mycotoxins especially concerning thermal treatments. High temperatures may cause, in fact, transformation or degradation of these compounds. This work is aimed to study the fate of mycotoxins during bakery processing, focusing on deoxynivalenol (DON) and deoxynivalenol-3-glucoside (DON3Glc), along the chain of industrial rusk production. Starting from naturally contaminated bran, we studied how concentrations of DON and DON3Glc are influenced by modifying ingredients and operative conditions. The experiments were performed using statistical Design of Experiment (DoE) schemes to synergistically explore the relationship between mycotoxin reduction and the indicated processing transformation parameters. All samples collected during pilot plant experiments were analyzed with an LC-MS/MS multimycotoxin method. The obtained model shows a good fitting, giving back relevant information in terms of optimization of the industrial production process, in particular suggesting that time and temperature in baking and toasting steps are highly relevant for minimizing mycotoxin level in rusks. A reduction up to 30% for DON and DON3Glc content in the finished product was observed within an acceptable technological range.

## 1. Introduction

Cereal and derived products are at the base of the human and animal diet [1]. Approximately 600 million tons of wheat are produced per year all over the world [2] and most of them are converted to wheat flour for human consumption and destined to bakery products. Exposure to toxic metabolites, which may occur in grains used for foodstuffs production, represents a food safety concern. In the last decade, researchers have focused their attention on the fate of these compounds during food processing that could affect the level found in finished products.

Trichothecene mycotoxins, which frequently occur in cereals as well as in cereal-based products, are secondary metabolites produced mainly by *Fusarium* ear blight pathogens, such as *Fusarium graminearum* or *F. culmorum.* These fungi are widely distributed in the temperate zone worldwide [3]. Deoxynivalenol (DON) is the most frequent *Fusarium* toxin in wheat in the more temperate regions of the world [4,5], and a wide range of cereal-based foods have been reported to be contaminated by this toxin [6,7].

Maximum tolerable limits have been set by the European Commission (EC) for this mycotoxin in food products: in particular, the maximum DON level under current legislation is 500 μg/kg in finished products such as bakery wares, pastries, or biscuits [8].

Food processing, including sorting, trimming, cleaning, dehulling, milling, brewing, cooking, baking, frying, roasting, canning, flaking, alkaline cooking, nixtamalisation, and extrusion, can have an impact on mycotoxin levels, but details of the effects remain mostly unclear [9]. Due to their thermal and chemical stability, mycotoxins can only be partly removed or redistributed by food processing procedures that may involve physical and chemical steps [10,11] so preventive measures should be adopted at all stages of the production chain to prevent contamination of final food products [12]. It is fundamental to better evaluate all the aspects of mycotoxin contamination starting from their level due to the pre-harvest fungal development, up to the effect of the transformation processes and the mycotoxin occurrence in the finished products.

The stability of DON, during various food processing practices and steps along the production chain, has been the target of several studies with controversial outcomes in some circumstances; for specific concerns of the milling phase there is in any case in this scientific literature a general alignment in terms of distribution of DON among the milling fractions in naturally *Fusarium* contaminated wheat. It is shown that the highest concentration of this mycotoxin remained in the bran layer, while the white flour the levels of DON were reasonably lower (compared with the whole grain). In particular, levels of DON in bran were two or more times higher than in wheat, indicating a concentration of toxin in the outer parts of the kernel [13,14,15,16]: therefore, the mycotoxin content could be higher in wholegrain bakery products.

The extent of DON reduction during thermal food processing operations seems to be quite variable and dependent on the processing conditions applied. Thermal treatment during bakery process reported variable effects on DON level: some authors suggested a reduction in the final contamination [17,18,19,20]; other researchers observed no reduction or increase at temperatures ranging from 170 to 350 °C [14,21,22,23]. The effect of the thermal treatment is strictly related to technological parameters, in particular, to the setting of the temperature/time conditions [24,25]. Basically, mycotoxin behavior is affected by the thermal treatment as well as the fermentation step [17], because of the yeast’s effect; up to now, data on the activity exerted by yeasts are rather contradictory with high stability of DON during the fermentation stage [26] or increase, probably due to the release of bound DON, as reported by Bergamini *et al.* [27]. This aspect has been also investigated by other researchers that suggested that the release is due to the activity of the enzymes of the improver agents and to the bacterial metabolism [28].

In recent years, the contribution of modified mycotoxins has been considered: mycotoxins might co-occur with structurally related compounds, generated by plants (so called “masked mycotoxins”) or by the technological processes [29]. These modified forms show a very different chemical behavior compared to the parent toxins. One of the most prominent resistance mechanisms is the detoxification of DON by conversion into deoxynivalenol-3-glucoside (DON3Glc), which is compartmentalized into the vacuoles or the cell walls [30,31]. Technological processes play an important role in this context, since reactions with macromolecular components such as sugars, proteins, or lipids can be induced upon treatments, while on the other hand parent toxins might be released from the conjugated forms. This mechanism could explain DON increase observed by several authors [14,27] within the fermentation phase of dough, as indicated above. Vidal *et al.* [32] studied the fate of DON and DON3Glc during bread making: DON increased from the unkneaded mix to fermented dough and decreased during baking, depending on the initial concentration in the flour. DON3Glc content increased both during kneading and fermentation as suggested by Zachariasova *et al.* [33], and also during baking, most likely due to the glycosylation of DON in the initial stages of baking before enzyme inactivation, in contrast with previous investigations conducted by Kostelanska *et al.* [34] and Simsek *et al.* [35].

Data show a clear health issue associated to mycotoxin ingestion and the need for regulating masked forms has been already recognized by European regulatory bodies, nevertheless, due to the lack of analytical methods, exposure and toxicological data, implementation remained vague until now.

In July 2013, the European Food Safety Authority (EFSA) accepted the mandate for a Scientific Opinion asked by the European Commission on the risks for animal and humans associated to the occurrence of DON and its derivatives [36,37].

Similarly, EFSA recently published a Scientific Opinion about the toxicological impact of modified mycotoxins other than DON [38]. Most recently, the European Commission pointed to the need to “analyze also the masked mycotoxins, in particular the mono- and di-glycosylated conjugates of T-2 and HT-2 toxin” in the current recommendation on indicative levels for the sum of T-2 and HT-2 in cereals and cereal-based foods [39].

The scope of the present research was to verify how DON and DON3Glc concentration is influenced by modifications of technological parameters and ingredients during the production of rusks. Rusk processing offers the opportunity to investigate the effects of a series of technological processes: fermentation, baking, and toasting.

Experiments were performed starting from naturally contaminated bran, obtained from wheat milling process, using a pilot-plant. A model was developed using statistical Design of Experiment (DoE) schemes for screening purposes to explore different factors with the aim to reveal whether they have an influence on the final mycotoxin contamination level. In our approach, we take into account only those modifications that could be actually applied at an industrial level in order to minimize mycotoxin levels in the finished product that has to be appreciable by the consumers from the organoleptic point of view.

## 2. Results and Discussion

The main objective of our approach was to explore the possible impact of process variables (factors) on DON concentration (response), in order to minimize the toxin content.

**Figure 1:**
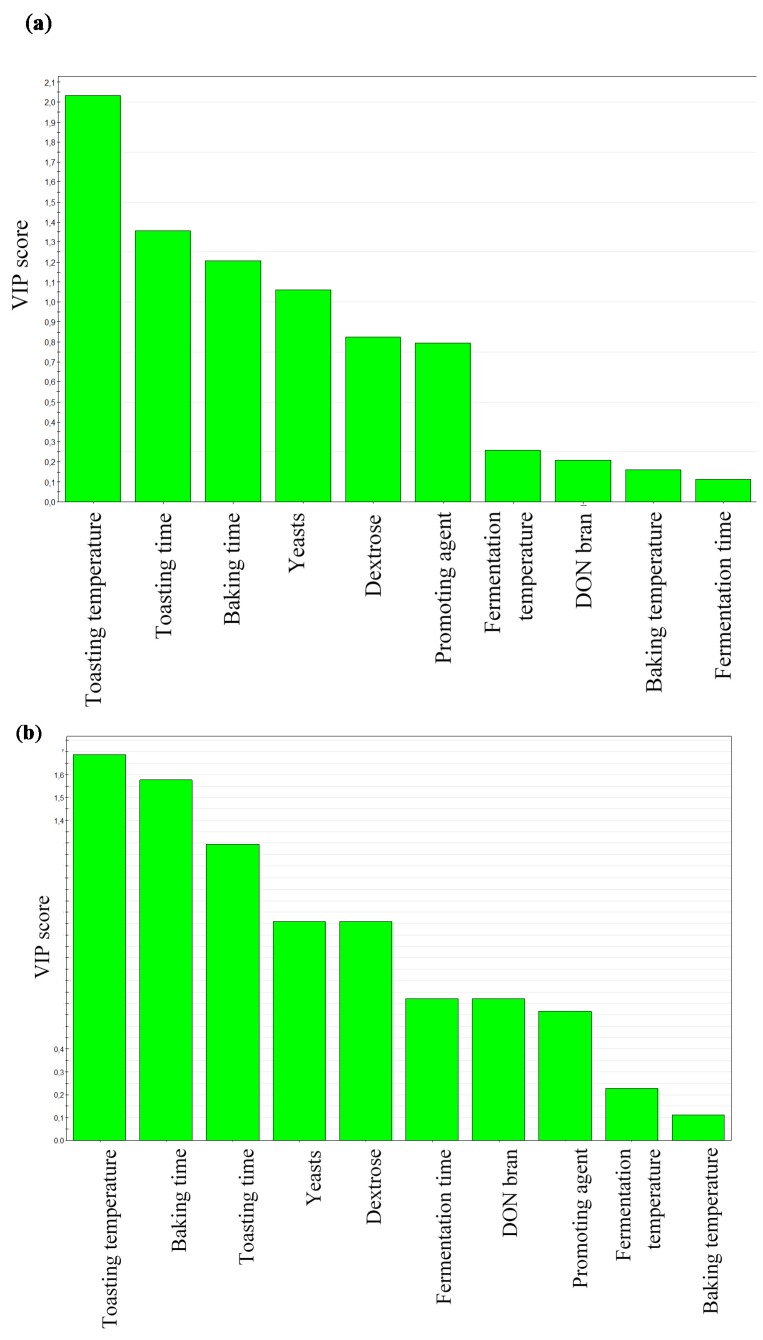
Variable Importance Plot (VIP) obtained from Design of Experiments rusk-making experiments (**a**) data referred to DON; (**b**) data referred to DON3Glc levels.

In this study, ten factors were taken into account (Table 1): DON contamination level of the starting bran, dextrose, yeast and enzyme amounts, fermentation time and temperature, baking time and temperature, and toasting time and temperature. For each parameter, a well-defined variation range was assessed, as reported in Table 2. The statistical model required 19 single experiments; in each experiment, the DON and DON3Glc levels were measured by LC-MS/MS in the mix flour/bran, before and after fermentation, after baking, and after toasting, as previously explained. After analyzing the sample sets of the chosen screening design, MODDE software was exploited to point out the influence of a particular factor and to calculate the regression coefficient in a correspondent developed polynomial model. All values were collected and computationally combined in a Variable Importance Plot (VIP, Figure 1), illustrating the importance of a variable to the measurements of a process/phenomenon, in order to better understand how each factor influences mycotoxin response. VIP is in fact the acronym for “variable importance plot” in the projection and it represents the most condensed way of expressing output as a weighed summary of all loadings and across all responses.

**Table 1:** Processing conditions during rusk experiments in the pilot-scale plant.

Treatment	Rusk Making		
	Minimum	Optimum	Maximum
DON bran level (μg/kg)	600	1050	1500
Dextrose (%)	2	4	6
Yeast (%)	2	3	4
Promoting agents (%)	0	0.5	1
Fermentation time (min)	40	50	60
Fermentation temperature (°C)	26	36	46
Baking time (min)	12	21	30
Baking temperature (°C)	180	195	210
Toasting time (min)	15	20	25
Toasting temperature (°C)	110	130	150

**Table 2:** Data set for screening variables effects on mycotoxin levels within the rusk-making process steps.

Experiment Number	DON in Bran (μg/kg d.m.) ^1^	Dextrose (%)	Yeast (%)	Promoting Agent (%)	Fermentation Stage		Baking Stage		Toasting Stage	
					Time (min)	Temperature (°C)	Time (min)	Temperature (°C)	Time (min)	Temperature (°C)
1	600 ± 16	2	2	0	40	26	12	180	25	150
2	1500 ± 92	2	2	0	60	26	30	210	15	110
3	600 ± 16	6	2	0	60	46	12	210	15	110
4	1500 ± 92	6	2	0	40	46	30	180	25	150
5	600 ± 16	2	4	0	60	46	30	180	15	150
6	1500 ± 92	2	4	0	40	46	12	210	25	110
7	600 ± 16	6	4	0	40	26	30	210	25	110
8	1500 ± 92	6	4	0	60	26	12	180	15	150
9	600 ± 16	2	2	1	40	46	30	210	15	150
10	1500 ± 92	2	2	1	60	46	12	180	25	110
11	600 ± 16	6	2	1	60	26	30	180	25	110
12	1500 ± 92	6	2	1	40	26	12	210	15	150
13	600 ± 16	2	4	1	60	26	12	210	25	150
14	1500 ± 92	2	4	1	40	26	30	180	15	110
15	600 ± 16	6	4	1	40	46	12	180	15	110
16	1500 ± 92	6	4	1	60	46	30	210	25	150
17	1050 ± 48	4	3	0.5	50	36	21	195	20	130
18	1050 ± 48	4	3	0.5	50	36	21	195	20	130
19	1050 ± 48	4	3	0.5	50	36	21	195	20	130

^1^ Data expressed as mean value ± standard deviation on dry matter basis (d.m.) of a final number of four replicates.

### DON and DON3Glc Levels during Rusk-Making

The replicated values of DON and DON3Glc reduction (calculated as the difference between DON and DON3Glc concentration in the initial flour-bran mix and in the final toasted rusks) in each experiment were averaged and used in the statistical elaboration. Data for DON and DON3Glc concentrations were expressed on dry matter, corrected according to the recipe formulation, and are reported in Table 3.

**Figure 2:**
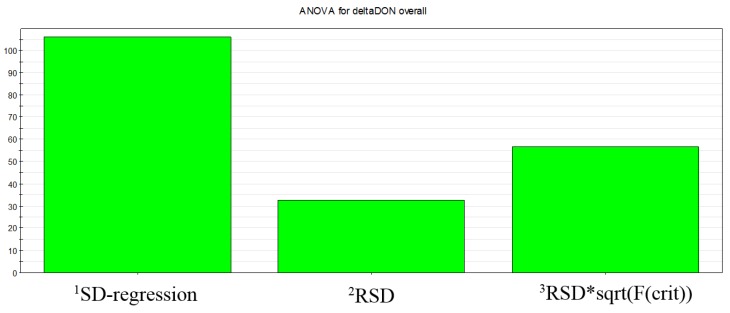
ANOVA plot obtained from Design of Experiments rusk-making experiments—data referring to DON levels. ^1^ *SD-regression*: it shows the variation of the response explained by the model, adjusted for degrees of freedom; ^2^ *RSD*: it shows the variation of the response not explained by the model, adjusted for degrees of freedom; ^3^ RSD*sqrt (*F*(crit)): it shows RSD (second bar) multiplied by the square root of the critical *F*; the critical *F* is the value of the F-distribution over which SD regression is statistically significant at the 95% confidence level.

Partial least-squares (PLS) was chosen for statistical regression treatment. The statistical model efficiency was evaluated by two main parameters: fitting (*R*^2^) and prediction (*Q*^2^) values. *R*^2^ measures the proportion to which the statistical model accounts for the variation of a given data set, while *Q*^2^ refers to its ability to generate prediction. Our statistical model gave a high fitting value (*R*^2^ > 0.8) and a good prediction value (*Q*^2^ > 0.6), referring to the MODDE software output settings. Model robustness was also confirmed by ANOVA plot (Figure 2) standard deviation of the regression being much larger than standard deviation of the residuals.

As shown in Figure 1a, a variable trend is recorded after the fermentation step, where DON is only slightly influenced by fermentation time and temperature but more affected by initial yeast amount in the recipe; this outcome differs from results obtained in other studies [14,27] in which DON increase was observed. A hypothesis that could justify some of the different findings in fermentation effects is the use of different “yeast mixtures” (*i.e.*, different strains of *Saccharomyces cerevisiae*, or mix with lactic acid bacteria).

**Table 3:** Design of experiments and corresponding analytical results for deoxynivalenol (DON) levels throughout rusk-making process.

Experiment Number	DON in Bran (μg/kg d.m.) ^1^	MC ^2^ (%)	DON in Dough (μg/kg d.m.) ^1^	MC ^2^ (%)	DON in Fermented Dough (μg/kg d.m.) ^1^	MC ^2^ (%)	DON in Baked Rusk (μg/kg d.m.) ^1^	MC ^2^ (%)	DON in Toasted Rusk (μg/kg d.m.) ^1^	MC ^2^ (%)
1	600 ± 16	12	273 ± 25	43	273 ± 29	43	239 ± 10	38	194 ± 16	3
2	1500 ± 92	12	349 ± 34	41	340 ± 25	40	334 ± 12	33	329 ± 7	10
3	600 ± 16	12	379 ± 21	42	333 ± 11	39	338 ± 5	36	322 ± 20	14
4	1500 ± 92	12	348 ± 30	41	359 ± 18	41	351 ± 23	33	199 ± 16	1
5	600 ± 16	12	259 ± 15	43	261 ± 12	42	246 ± 12	32	188 ± 20	5
6	1500 ± 92	12	392 ± 30	41	339 ± 26	41	360 ± 15	37	317 ± 11	5
7	600 ± 16	12	257 ± 16	44	242 ± 10	44	199 ± 24	33	172 ± 28	4
8	1500 ± 92	12	306 ± 12	41	300 ± 5	41	324 ± 15	37	239 ± 15	5
9	600 ± 16	12	242 ± 18	44	210 ± 22	42	198 ± 15	33	165 ± 26	4
10	1500 ± 92	12	339 ± 31	44	333 ± 15	44	343 ± 12	36	315 ± 10	6
11	600 ± 16	12	213 ± 14	39	220 ± 18	38	229 ± 19	38	200 ± 11	5
12	1500 ± 92	12	318 ± 27	43	298 ± 32	42	347 ± 36	38	290 ± 15	2
13	600 ± 16	12	253 ± 12	40	256 ± 20	40	243 ± 20	38	149 ± 19	6
14	1500 ± 92	12	339 ± 31	39	311 ± 23	38	330 ± 18	37	308 ± 12	4
15	600 ± 16	12	211 ± 18	40	215 ± 26	40	253 ± 3	38	212 ± 5	6
16	1500 ± 92	12	280 ± 14	41	313 ± 21	43	197 ± 32	29	68 ± 11	1
17	1050 ± 48	11	219 ± 10	45	229 ± 19	44	225 ± 21	38	194 ± 16	5
18	1050 ± 48	11	272 ± 8	45	248 ± 30	44	225 ± 19	38	196 ± 12	5
19	1050 ± 48	11	251 ± 20	45	213 ± 19	44	218 ± 24	38	186 ± 21	5

^1^ Data expressed as mean value ± standard deviation on dry matter basis (d.m.) of a final number of four replicates; ^2^ MC: Moisture Content.

Coming now to consider the thermal influence, in the present case, VIP would suggest that the evolution of DON appears to be significantly affected by the baking time, toasting time, and temperature: among these factors, the statistical analysis suggests that baking temperature has a less important effect than baking time on DON stability, according to what is reported by previous studies [26,32]. The lower effect of baking temperature compared to baking time can be explained taking into consideration the heating gradient occurring in the loaf during baking. In this case, temperature in the core of the loaf is about 100 °C, independently of the oven temperature.

The fermentation phase, as said above, together with the other involved parameters, had a smaller or negligible effect on final DON concentration. This is in agreement with another research work conducted by Suman *et al.* [40] on cracker-making and a reasonable explanation can be the following: taking into account the overall processes and correspondent technological parameters, the influence of heat-exchange phenomena is crucial. Rusks undergo to two subsequent heating treatments (baking the entire loaf and then toasting after having cut the loaf into several slices) and both crackers and rusks (in the baking and toasting phases, respectively) have a significantly higher heat-exchange surface with respect to bread.

One-way analysis of variance was carried out by applying Turkey’s post hoc test (*p* < 0.05) using SPSS statistics 21.0 software (SPSS Inc., Chicago, IL, USA, 2003): after baking and toasting, a decrease from 8% to 19% and from 19% to 65% in DON levels, respectively, were observed and this phenomenon indicates probable degradation, due to the thermal treatment at high temperature. A potential reduction within the total processing up to 30% may be feasible: the opportune increase of the toasting time/temperature can determine such a reduction of the free DON concentration in the final rusks, remaining in an acceptable range of technological and organoleptic conditions.

As a naturally contaminated raw material was used for rusk production, other mycotoxins can co-occur in the final products: DON3Glc was observed within a range of about 5%–15% of the DON concentration, similarly to what we found in a previous work [41]. The experimental design reported herein was planned taking into consideration only DON contamination in bran, while DON3Glc co-occurrence was found later. For this reason, DON3Glc concentration was not significantly different in the three initial batches, and it cannot be considered as a factor for the statistical evaluation. However, although its average concentration is relatively low (32 ± 2 μg/kg), data are still significant in term of corresponding uncertainties (Table 4) and some indication can be obtained as well. In particular, an increase up to 48% can be envisaged after the fermentation step, followed by a reduction during baking and toasting, as already reported by other studies [34,35]. As a result of the overall variability of the statistical approach, the final concentration seems to be affected only by three factors: toasting time, temperature, and baking time, as reported in Figure 1b.

Considering the modelling of DON and DON3Glc evolution during rusk-making, it can be reasonably assumed that toasting and baking stages support toxin degradation.

**Table 4:** Design of experiments and corresponding analytical results for deoxynivalenol-3-glucoside (DON3Glc) levels throughout rusk-making process.

Experiment Number	D3Glc Dough (μg/kgd.m.) ^1^	MC ^2^ (%)	D3Glc Fermented Dough (μg/kg d.m.) ^1^	MC ^2^ (%)	D3Glc Baked Rusk (μg/kg d.m.) ^1^	MC ^2^ (%)	D3Glc Toasted Rusk (μg/kg d.m.) ^1^	MC ^2^ (%)
1	29 ± 6	43	29 ± 5	43	22 ± 6	38	11 ± 5	3
2	77 ± 10	41	77 ± 9	40	27 ± 5	33	23 ± 3	10
3	36 ± 3	42	41 ± 4	39	30 ± 3	36	25 ± 4	14
4	46 ± 7	41	64 ± 9	41	31 ± 4	33	6 ± 1	1
5	23 ± 4	43	22 ± 5	42	18 ± 2	32	12 ± 3	5
6	60 ± 6	41	64 ± 7	41	34 ± 5	37	21 ± 4	5
7	18 ± 3	44	18 ± 4	44	8 ± 1	33	6 ± 2	4
8	39 ± 4	41	45 ± 6	41	23 ± 3	37	18 ± 3	5
9	23 ± 2	44	29 ± 3	42	12 ± 2	33	7 ± 0	4
10	54 ± 5	44	65 ± 7	44	32 ± 4	36	27 ± 6	6
11	19 ± 3	39	17 ± 2	38	14 ± 3	38	10 ± 2	5
12	41 ± 5	43	43 ± 3	42	30 ± 4	38	20 ± 5	2
13	26 ± 2	40	29 ± 2	40	19 ± 3	38	8 ± 4	6
14	41 ± 2	39	61 ± 5	38	28 ± 3	37	20 ± 5	4
15	17 ± 3	40	19 ± 2	40	14 ± 2	38	10 ± 3	6
16	30 ± 4	41	40 ± 3	43	9 ± 1	29	<0.1	1
17	17 ± 3	45	30 ± 5	44	16 ± 4	38	9 ± 1	5
18	11 ± 5	45	19 ± 2	44	15 ± 3	38	8 ± 2	5
19	17 ± 3	45	24 ± 4	44	14 ± 5	38	9 ± 1	5

^1^ Data expressed as mean value ± standard deviation on dry matter basis (d.m.) of a final number of four replicates; ^2^ MC: Moisture Content.

## 3. Experimental Section

### 3.1. Chemicals

Methanol and acetonitrile (both LC gradient grade) were purchased from J.T. Baker (Deventer, The Netherlands), while ammonium acetate (MS grade) and glacial acetic acid (p.a.) were obtained from Sigma-Aldrich (Vienna, Austria). Water was purified successively by reverse osmosis and a Milli-Q plus system from Millipore (Molsheim, France). Mycotoxin standards were obtained from the following commercial sources: Romer Labs (Tulln, Austria), Sigma-Aldrich (Vienna, Austria), Iris Biotech GmbH (Marktredwitz, Germany), Axxora Europe (Lausanne, Switzerland) and LGC Promochem GmbH (Wesel, Germany). Standards were dissolved in acetonitrile (ACN) if not stated otherwise. DON3Glc was isolated from wheat treated with DON [31]. All solutions were stored at −20 °C and were brought to room temperature before use.

### 3.2. Raw Material Selection and Preparation

For the baking experiments, different batches of wheat bran naturally infected with *Fusarium* spp. were analyzed with a focus on DON contamination levels. Among the analyzed batches, three bran batches were established, according to DON contamination level and technological characteristics. In particular, three different levels of DON contamination were selected (bran A, 600 ± 16 μg/kg; bran B, 1050 ± 48 μg/kg; bran C, 1500 ± 92 μg/kg).

Industrial wholegrain flours (mix A, mix B and mix C) ready for the experiments were obtained by mixing the contaminated bran (percentage 7%–10% as maximum) with common wheat flours that present a contamination content of about 150–300 μg/kg (depending on the lots); water was then added during dough preparation: 40%–45% with respect to the total weighed dough. Therefore, at the end of these steps, the different final doughs achieved the final concentration of DON and DON3Glc specified in Table 3 and 4, respectively.

### 3.3. Moisture Content Determination

The moisture contents of mix flours, doughs, and baked and toasted products were measured by taking a 5 g ground sample and heating it in a thermostatic oven at 105 °C for over 6 h. All the results were calibrated and compared on a dry matter basis.

### 3.4. Experimental Design

The DoE method is used in many industrial sectors in the development and optimization of manufacturing process, making a set of experiments representative with regard to a given question. One of the main types of DoE-application is the screening and identification of important factors (the “treatments”): initial screening experimental designs are used to locate the most fruitful part of the experimental region. The main objective is to explore many factors in order to reveal whether they have an influence on the responses allowing to extract an answer with regard to the influence of an important factor. Information is also gained about how to modify the settings of the important factors, to possibly further enhance the result [42].

In the present study different parameters of the transformation processes were varied during the experiments. Three replicates of the central point were performed to estimate the experimental error. Among the rusk-making parameters, several conditions were varied during the experiments: DON contamination level on wheat bran, levels of dextrose, levels of yeast (*Saccharomyces cerevisiae*) and rusk-making promoting agents content (as percentage in recipe), fermentation time and temperature, baking time and temperature as well as toasting time and temperature. Each selected treatment was varied within a range defined according to the technological requirements to obtain an organoleptically appreciable product for consumers: the central experimental values, indicated in the Table 1 as “optimum”, represent the optimal combination of ingredients/recipe and operative conditions that permit achievement of the better finished product.

Experimental data were then analyzed by a multi-variate analysis approach based on the partial least-squares (PLS) technique, using a dedicated statistical package (MODDE software, version 9.1; Umetrics, Umea, Sweden).

### 3.5. Dough Preparation and Rusk-Making

**Figure 3:**
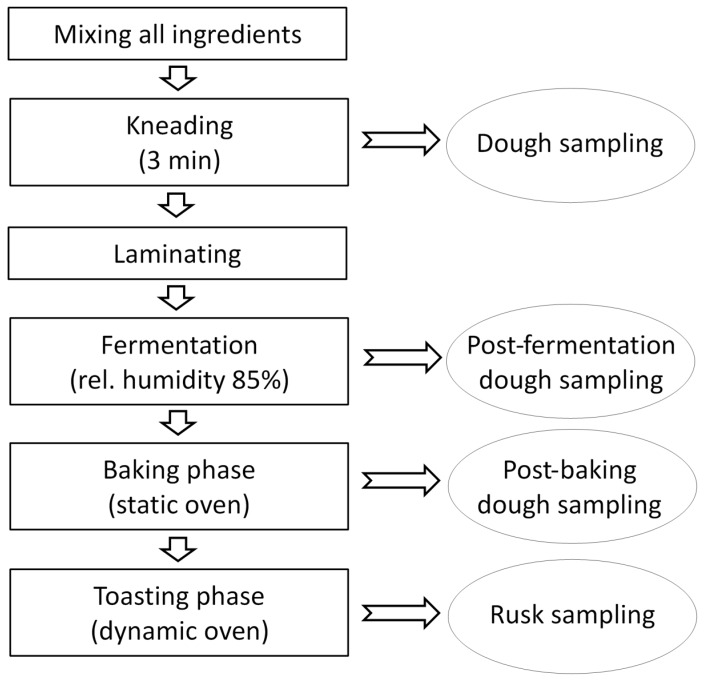
Scheme of the rusk-making process.

Different doughs were prepared starting from wheat bran and standard wheat flour (mix A, mix B and mix C) for obtaining a final loaf of about 800 g; the ingredients were wheat flour, bran, fractionated palm oil, salt. Yeast (*S. cerevisiae*), rusk-making promoting agents (enzymes) and dextrose were added as indicated in experimental design model. The optimal amount of water to be added to each dough sample was established on the basis of previous experiments and internal technological knowledge. The process consists substantially of the following steps: firstly, all ingredients were mixed using a test planetary kneader for 3 min and dough was left to rest for 5 min at room temperature. Dough was shaped (laminating step) and fermented at different conditions in a leavening cell (FermaLievita Alaska, Bologna, Italy) at a relative humidity (RH%) of 85%. Baking was performed in a pilot-scale static oven (Tagliavini, Parma, Italy), while toasting was performed in a pilot-scale dynamic oven (Tagliavini, Parma, Italy). The overall rusk-making process is summarized in Figure 3. Since the experimental plan involved randomized changes in ingredient amounts and in the condition of rusk fermentation, baking and toasting, 19 different tests for the pilot-plant were performed (Table 2) according to the MODDE outputs. Four identical doughs were used for each realization of the experiment, as the mycotoxin content was examined in the mix flour and bran, before the fermentation step, after the fermentation step, after the baking process, and after the toasting process. Before mycotoxin analysis, samples were stored at −20 °C. Extraction was performed in duplicate.

### 3.6. Sample Extraction

Sample preparation was carried out according to Sulyok *et al.* [43] with slight modifications. In brief, 5.00 ± 0.01 g of the ground sample were extracted with 20 ml acetonitrile/water/acetic acid (79/20/1, *v*/*v*/*v*) for 90 min using a GFL 3017 rotary shaker (GFL, Burgwedel, Germany) and subsequently, centrifuged for 2 min at 3000 rpm (radius 15 cm) on a GS-6 centrifuge (Beckman Coulter Inc., Fullerton, CA, USA). Then, the extract was transferred into glass vials using Pasteur pipettes, and a 350 μL aliquot was diluted with the same volume of dilution solvent (acetonitrile/water/acetic acid 20/79/1, *v*/*v*/*v*). After appropriate mixing, 5 μL of the diluted extract were injected into the LC-MS/MS system without further pre-treatment.

### 3.7. Instrumental Conditions

Detection and quantification was performed with a QTrap 5500 MS/MS system (Applied Biosystems, Foster City, CA, USA) equipped with a TurboV electrospray ionization (ESI) source and a 1290 Series UHPLC system (Agilent Technologies, Waldbronn, Germany). Chromatographic separation was performed at 25 °C on a Gemini^®^ C18-column, 150 × 4.6 mm i.d., 5 μm particle size, equipped with a C18, 4 × 3 mm i.d. security guard cartridge (all from Phenomenex, Torrance, CA, USA). Elution was carried out in binary gradient mode. Both mobile phases contained 5 mM ammonium acetate and they were composed of methanol/water/acetic acid 10:89:1 (*v*/*v*/*v*; eluent A) and 97:2:1 (*v*/*v*/*v*; eluent B), respectively. After an initial time of 2 min at 100% A, the proportion of B was increased linearly to 50% within 3 min. Further linear increase of B to 100% within 9 min was followed by a hold-time of 4 min at 100% B and 2.5 min column re-equilibration at 100%. The flow rate was 1 mL/min. The column effluent was transferred via a six-port valve (VICI Valco Instruments, Houston, TX, USA) either to the mass spectrometer (between 2 and 17 min; no flow splitting was used) or to the waste. ESI-MS/MS was performed in the scheduled multiple reaction monitoring (sMRM) mode both in positive and negative polarities in two separate chromatographic runs per sample with the following settings: source temperature 550 °C, curtain gas 30 psi (69 kPa of max. 99.5% nitrogen), ion source gas 1 (sheath gas) 80 psi (345 kPa of nitrogen), ion source gas 2 (drying gas) 80 psi (345 kPa of nitrogen), ion spray voltage −4.500 V and +5.500 V, respectively, collision gas (nitrogen) medium. The sMRM detection window of each analyte was set to the respective retention time ±27 s and ±42 s in positive and in negative mode, respectively. The target scan time was set to 1 s. The optimization of the analyte-dependent MS/MS parameters was performed via direct infusion of standards (diluted in a 1:1 mixture of eluent A and B) into the MS source using a 11 Plus syringe pump (Harvard Apparatus, Holliston, MA, USA) at a flow rate of 10 μL/min for the corresponding values. Confirmation of positive analyte identification is obtained by the acquisition of two sMRMs per analyte, which yields 4.0 identification points according to Commission Decision [44]. Retention times for DON and DON3Glc are 6.12 min and 5.99, respectively. The mean LOD and LOQ for the tested matrices are 0.15 μg/kg and 0.20 μg/kg for DON, respectively, and 0.04 μg/kg and 0.10 for DON3Glc, respectively. Further method information can be found in Malachova *et al.* [45]. Performance characteristics of the method were verified for the studied matrices (mix flour/bran, dough, baked and toasted rusk) prior to analysis. Spiking experiments were performed on flour, dough, baked, and toasted rusk. The selected levels for DON and DON3Glc were 200 μg/kg and 20 μg/kg, respectively. Three replicates were conducted for each matrix and spiking level. The mean recovery for DON was 106% ± 4% in flour, 103% ± 2% in dough, 113% ± 1% in baked rusk and 91% ± 11% in toasted rusk. Concerning DON3Glc recovery, the mean values were 100% ± 10% in flour, 75% ± 5% in dough, 80% ± 3% in baked rusk and 98% ± 2% in toasted rusk. Data were corrected for recovery percentage.

## 4. Conclusions

The influence exerted by a set of rusk-making parameters on DON and DON3Glc level in wholegrain rusks was investigated using naturally contaminated bran over an industrial pilot-scale production line.

Along the production process, DON seems to be strongly affected in particular by the baking time and toasting time and temperature, with a parallel negligible effect determined by the fermentation phase.

Exploiting DoE approach, the obtained model provided interesting information about the trends and directions useful to be followed exactly in this industrial perspective: increasing in particular time/temperature in the toasting phase, even staying in an acceptable technological range, could reduce DON and DON3Glc content in rusks up to 30%.

As pointed out by this study, a careful control of the food processing steps may actually lead to a minimization of mycotoxin content in the final product, providing a powerful synergistic strategy to be combined to good agricultural practices for reducing consumers exposure.
